# Stimulation of natural killer cells with rhCD137 ligand enhances tumor-targeting antibody efficacy in gastric cancer

**DOI:** 10.1371/journal.pone.0204880

**Published:** 2018-10-15

**Authors:** Toshihiro Misumi, Kazuaki Tanabe, Nobuaki Fujikuni, Hideki Ohdan

**Affiliations:** Department of Gastroenterological and Transplant Surgery, Applied Life Sciences, Institute of Biomedical and Health Sciences, Hiroshima University, Hiroshima, Japan; Universita degli Studi di Parma, ITALY

## Abstract

Although many anticancer agents for gastric cancer have been developed, the prognosis for many patients remains poor. Recently, costimulatory immune molecules that reactivate antitumor immune responses by utilizing the host immune system have attracted attention as new therapeutic strategies. CD137 is a costimulatory molecule that reportedly potentiates the antitumor activity of tumor-targeting monoclonal antibodies (mAbs) by enhancing antibody-dependent cellular cytotoxicity. However, it remains unclear whether CD137 stimulates tumor-regulatory activity in gastric cancer. In this study, we investigated the antitumor effects of CD137 stimulation on gastric cancer cells administered tumor-targeting mAbs. Our results showed that human natural killer (NK) cells were activated by expressing CD137 after encountering trastuzumab-coated gastric cancer cells, and that stimulation of activated NK cells in the presence of trastuzumab and recombinant human CD137 ligand (rhCD137L) enhanced cytotoxicity and release of cytokines (IFN-γ, TNF, granzyme A, or granzyme B) as compared with activated NK cells with trastuzumab alone (*p* < 0.05). By combination treatment with rhCD137L, similar effects were obtained regarding cancer cell cytotoxicity in the presence of cetuximab (*p* < 0.01). Moreover, we revealed that CD137 expression was dependent upon the affinity between the Fc portion of the antibodies and FcγRIIIa of NK cells based on results indicating that human IgG1 and IgG3 subclasses enhanced CD137 expression (*p* < 0.001). These results confirmed that FcγRIIIA polymorphisms (158 V/V) enhanced CD137 expression to a greater degree than 158 F polymorphisms (*p* = 0.014). Our results suggested that CD137 stimulation could promote the effects of tumor-targeting mAbs in gastric cancer, and that further investigation of antibody binding affinity and *in vivo* activities might improve therapeutic strategies related to the treatment of gastric cancer patients.

## Introduction

Gastric cancer remains the fifth most common malignancy and the third leading cause of cancer death worldwide [1]. Although its global incidence is declining, it remains highly prevalent in Asian countries, such as China, Korea, and Japan [1, 2]. The prognosis of patients with gastric cancer has been improved by early detection and surgical resection with regional lymphadenectomy; however, the mortality associated with advanced gastric cancer remains high and is mainly a result of recurrence and metastasis. The expected survival period of untreated stage IV gastric cancer is reportedly 3 to 5 months, and systemic chemotherapy alone has been reported to extend overall survival by up to 9 to 13 months [3–5]. However, these results have been mostly unsatisfactory, and more active treatment strategies are required to improve outcomes for gastric cancer patients.

Tumor-targeting antibodies are among the most important developments in the field of cancer therapy in the last 20 years. Trastuzumab, a humanized monoclonal antibody (mAb) targeting human epidermal growth factor receptor 2 (HER2), represents a type of chemotherapy that is now a standard approach for patients with HER2-positive advanced gastric cancer. Its antitumor effects involve the direct inhibition of the HER2-mediated signaling pathway and also the induction of antibody-dependent cell-mediated cytotoxicity (ADCC) via activated natural killer (NK) cells [6–8]. Trastuzumab reportedly prolonged median overall survival by 13.8 months for strongly HER2-positive patients in a randomized clinical trial [9]. However, many patients are unable to experience the benefits of trastuzumab treatment, because the HER2-overexpression rate [immunohistochemistry (IHC) score: 2+; fluorescent *in situ* hybridization-positive or IHC score: 3+] in gastric cancer patients ranges only from 12.1% to 15.6% [9, 10]. Therefore, many different strategies, such as combinations of oncolytic viruses, toll-like receptor (TLR) agonists, and engineered mAbs, are under development to stimulate immune effector cells implicated to promote ADCC [11–13]. In particular, selective targeting of activated NK cells might constitute an attractive strategy to improve ADCC without systemic toxicity associated with global NK cell stimulation, which is observed following systemic interleukin (IL)-2 or IL-12 administration [14, 15].

A recent study reported that CD137 (also known as 4-1BB), a surface glycoprotein belonging to the tumor-necrosis factor receptor superfamily, is upregulated on human NK cells when they encounter antibody-bound tumor cells, and that the cytotoxicity of these activated NK cells is enhanced by their exposure to an agonistic mAb against CD137, leading to improved antitumor activity in xenotransplant models of B cell lymphoma, breast, colon, and head and neck cancer [16–18]. However, it remains unclear whether CD137 stimulation can regulate tumor activity in gastric cancer. This study examined the ability of CD137 stimulation to enhance the anticancer efficacy of ADCC in gastric cancer via tumor-targeting mAbs, including trastuzumab. Additionally, we investigated the possibility for CD137 modulation dependent upon the binding affinity between NK cells and mAbs.

## Materials and methods

### Cell lines and culture

The human gastric cancer cell line TMK-1 was kindly provided by Dr. E. Tahara (Hiroshima University, Hiroshima, Japan). The NCI-N87 cell line was obtained from American Type Culture Collection (Manassas, VA, USA). GLM-5S and GLM-4 cells were kindly provided by Dr. H. Nakanishi (Aichi Cancer Center, Nagoya, Japan), and MKN-1 and MKN-45 cells were obtained from the Japanese Collection of Research Bioresources Cell Bank (Osaka, Japan). The GLM-5S and GLM-4 cells were cultured in Dulbecco’s modified Eagle medium (DMEM; Gibco, Grand Island, NY, USA) on collagen wells, and other gastric cell lines were cultured in Roswell Park Memorial Institute (RPMI) medium, all supplemented with 10% heat-inactivated fetal bovine serum (FBS; Sanko Chemical Co., Tokyo, Japan), 100 U/mL penicillin, and 100 μg/mL streptomycin (Gibco). Cells were grown as adherent cultures at 37°C in 5% CO_2_ and passaged after detachment by 0.05% trypsin.

### Therapeutic antibodies and proteins

Recombinant human CD137 ligand (rhCD137L) was obtained from PeproTech (Rocky Hill, NJ, USA). Trastuzumab and rituximab were obtained from Chugai Pharmaceutical Co. (Tokyo, Japan), and cetuximab was obtained from Merck Serono Co. (Tokyo, Japan). Pertuzumab was obtained from Genentech (San Francisco, CA, USA). IgG subclass mAbs (huIgG1/G2/G3/G4) were obtained from PeproTech.

### Antibodies for flow cytometry

Flow cytometric analyses were performed using a FACS Canto II cytometer (BD Biosciences, San Jose, CA, USA) to assess the purity and CD137-mediated expression of NK cells. The following mAbs were used for staining human peripheral blood mononuclear cells (PBMCs): phycoerythrin (PE)-conjugated CD56, PE CD137L, PE NKp30, PE NKp44, PE NKp46, PE CD122, PE CD117, PE TRAIL, PE CD107a, PE CD158e, PE CCR5, PE CD25, PE CD226, PE NKG2D, PE FasL, PE NKG2A, PE NKG2C, fluorescein isothiocyanate (FITC)-conjugated CD3, FITC CD158a, FITC CD158b, allophycocyanin (APC)-conjugated CD137, APC anti-HER2, APC anti-epidermal growth factor receptor (EGFR), and 7-aminoactinomycin D (7-AAD); all mAbs were obtained from BD Biosciences. Stained cells were collected on a FACS Canto II cytometer (BD Biosciences), and data were analyzed using FlowJo version X software (FlowJo, LLC, Ashland, OR, USA).

### CD137 expression on human NK cells from healthy individuals

PBMCs isolated from healthy individuals and purified NK cells isolated by negative magnetic cell sorting using NK cell-isolation beads (Miltenyi Biotec, Bergisch Gladbach, Germany) were obtained as described previously [19] at more than 90% purity, as defined by CD3^-^CD56^+^ and confirmed by flow cytometry. This study was conducted with informed consent using a protocol approved by the institutional review board of Hiroshima University Hospital (No. Hi-202). Purified NK cells were cultured with gastric cancer cell lines at a 1:1 ratio in the presence of trastuzumab (10 μg/mL) or rituximab (10 μg/mL) for 24 h. CD137 expression on NK cells was confirmed by flow cytometry. Assessment of CD137 on NK cells was performed in triplicate for each sample from five healthy individuals.

### *In vitro* NK cell functional assays

NK cell cytotoxicity was measured using a chromium-release assay. Target tumor cells were labeled with 100 μCi ^51^Cr per 1 × 10^6^ cells for 1.5 h, and purified NK cells were added at variable effector/target-cell ratios from 4:1 to 1:4. The lysis percentage was determined after 18 h of culture in the following three conditions: medium alone, trastuzumab (10 ng/mL), or trastuzumab (10 ng/mL) with rhCD137L (250 ng/mL). All assays were performed in triplicate with three independent NK cell samples.

To evaluate CD107a mobilization and cytokine release, purified NK cells were cultured with GLM-4 or MKN-1 cells. First, CD107a mobilization was assayed to evaluate NK cell degranulation. Purified NK cells were cultured with gastric cancer cells at a ratio of 1:1, with medium alone, trastuzumab (10 ng/mL), rhCD137L (250 ng/mL), or trastuzumab (10 ng/mL) with rhCD137L (250 ng/mL). After 24 h, CD56/CD107a double staining was carried out for analysis by flow cytometry. Phenotypes of NK cells in those conditions were also confirmed by flow cytometry with staining of NKp30, NKp44, NKp46, CD122, CD117, TRAIL, CD107a, CD158e, CCR5, CD25, CD226, NKG2D, FasL, NKG2A, NKG2C, CD158a, or CD158b.

Under conditions similar to those detailed above for NK cell degranulation, cell supernatants were analyzed for release of cytokines [human interferon (IFN)-γ, tumor necrosis factor (TNF), granzyme A, or granzyme B] using cytometric bead array (BD Biosciences) according to the manufacturer’s instructions. Cetuximab-focused *in vitro* assays were also performed under the same conditions in the presence or absence of rhCD137L. *In vitro* assays, CD107a mobilization, and cytokine assessment were performed with five independent samples.

### CD137 expression on NK cells in combination with two types of HER2 mAbs and *in vitro* cytotoxicity assay

Purified NK cells were cultured with the same proportion of GLM-4 cells in DMEM plus different concentrations of trastuzumab and pertuzumab (0–10 μg/mL) for 24 h. CD137 expression on NK cells was confirmed by flow cytometry. NK cell cytotoxicity was measured using a chromium-release assay in the following five conditions: medium alone, trastuzumab (5 ng/mL), pertuzumab (5 ng/mL), trastuzumab (5 ng/mL) and pertuzumab (5 ng/mL), or trastuzumab (5 ng/mL) and pertuzumab (5 ng/mL) with rhCD137L (250 ng/mL). All assessments were performed in triplicate for each of three samples from healthy individuals.

### CD137 expression on NK cells with the immobilized IgG subclass

Purified NK cells were plated at a concentration of 4.0 ×10^4^ cells/well in a 96-well flat-bottomed plate in the presence of either pre-coated (immobilized) or soluble monoclonal human IgG subclass (huIgG1/G2/G3/G4) at a concentration of 2 mg/mL. After 24 h, NK cells were harvested and analyzed for cell-surface CD137 expression by flow cytometry. Assessment of CD137 presentation on NK cells was performed in triplicate for each of five samples from healthy individuals.

### Analysis of the FcγRIIIa genotype

Genomic DNA was extracted from the PBMCs of 38 healthy individuals using Wizard SV genomic DNA purification system (Promega, Madison, WI, USA). Genotyping of FcγRIIIa 158 F/V (rs396991) was performed by polymerase chain reaction restriction-fragment-length polymorphism analysis as described previously [20]. CD137 expression on NK cells was compared between FcγRIIIa 158 V/V- and 158 F-carrier allele groups by flow cytometry.

### Statistical analysis

Statistical significance at the 95% confidence interval was analyzed using the unpaired Student’s *t* test. The correlation between HER2-expressing cells and CD137 upregulation of NK cells was analyzed by the Spearman correlation test. All statistical data were analyzed with Microsoft Excel (Microsoft Corporation, Redmond, WA, USA) and JMP 11.2.0 (SAS Institute Inc., Cary, NC, USA).

## Results

### Trastuzumab induces CD137 upregulation on human NK cells following incubation with HER2-positive tumor cells

**Fig 1:**
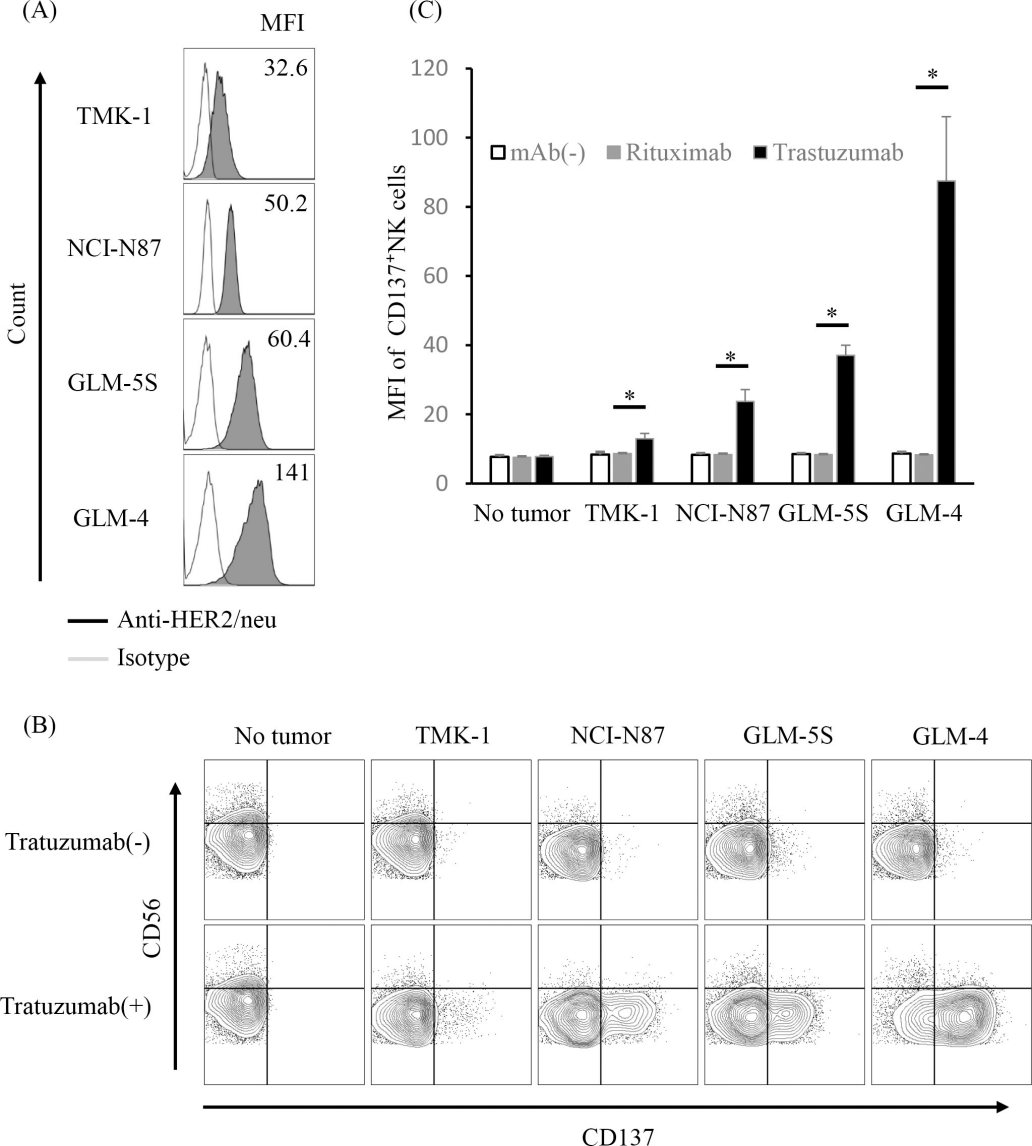
CD137 expression on NK cells following incubation of gastric cancer cells with trastuzumab. Purified NK cells from PBMCs of healthy individuals were analyzed for CD137 expression after 24-h culture with gastric cancer cell lines and trastuzumab. (A) HER2 expression on gastric cancer cell lines (TMK-1, NCI-N87, GLM-5S, and GLM-4). (B) CD137 expression on CD3^-^CD56^+^ NK cells from a representative healthy individual after 24-h culture with the respective gastric cancer cell lines in the presence or absence of trastuzumab. (C) Mean fluorescence intensity (MFI) of CD137 expression on NK cells from five healthy individuals cultured with the respective gastric cancer cell lines, * *p* < 0.001. Data are shown as the mean ± SEM.

Purified NK cells from healthy human patients were incubated with various HER2-expressing gastric cancer cell lines (Fig 1A) in the presence of trastuzumab or rituximab. These cell lines showed evidence of neither CD137 ligand nor the CD137 receptor (S1 Fig). After a 24-h incubation, we observed minimal changes in CD137 expression on NK cells incubated with cancer cell lines alone. By contrast, incubation with the combination of trastuzumab and gastric cancer cell lines resulted in significantly upregulated CD137 expression and presentation on NK cells compared with the combination of rituximab and gastric cancer cell lines. Especially, the enhancement of CD137 expression was dependent upon the degree of HER2 expression in gastric cancer cell lines (Spearman correlation test; *p* < 0.001) (Fig 1B and 1C).

### rhCD137L increases trastuzumab-mediated NK cell cytotoxicity

**Fig 2:**
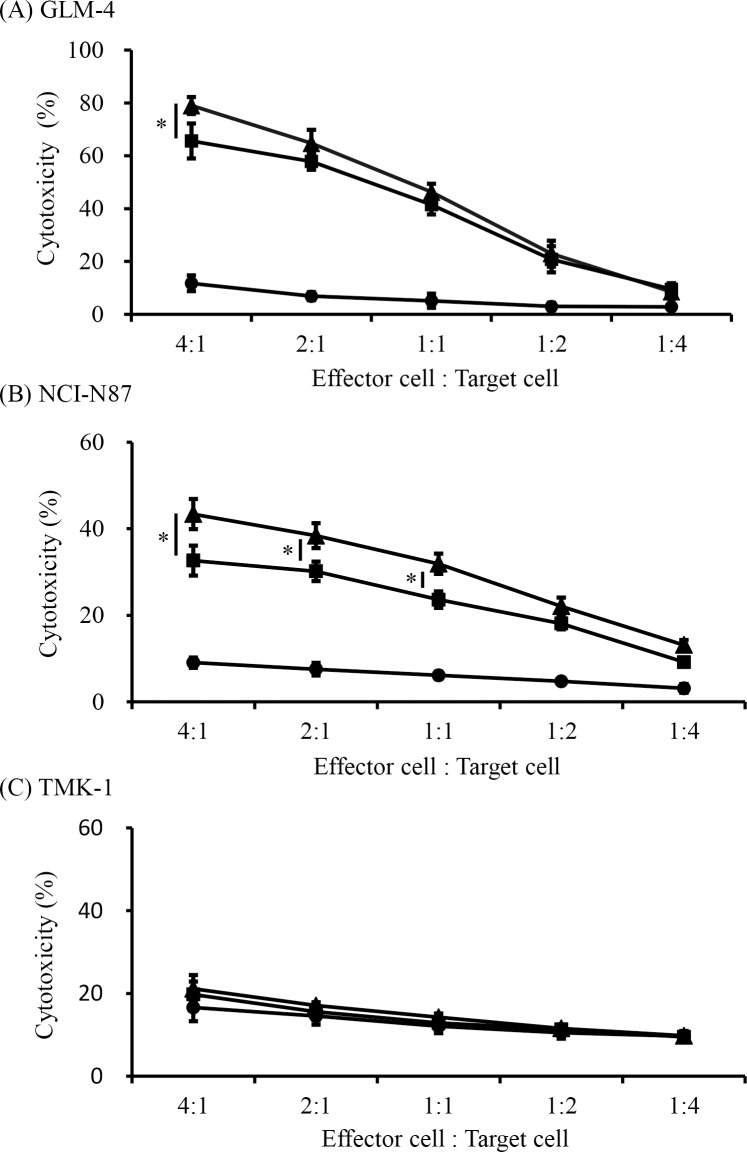
Enhancement of trastuzumab-mediated NK cell cytotoxicity by rhCD137L. To evaluate NK cell cytotoxic function, purified NK cells from three healthy individuals were incubated with chromium-labeled tumor cells, including (A) GLM-4, (B) NCI-N87, and (C) TMK-1 for 18 h with medium alone, trastuzumab (10 ng/mL), or trastuzumab plus rhCD137L (250 ng/mL). The lysis percentages of the target cells cultured with medium alone (circles), trastuzumab (squares), or trastuzumab plus rhCD137L (triangles) according to chromium release at varying effector (NK cells):target-cell ratios are shown. (A, *p = 0.03; B, *p < 0.01; C, *p* = not significant). Data are shown as the mean ± SEM.

To determine whether CD137 is a potential therapeutic target for enhancing NK cell function against gastric cancer, we investigated whether rhCD137L could enhance trastuzumab-mediated NK cell cytotoxicity (Fig 2). NK cells from healthy individuals were cultured with trastuzumab and ^51^Cr-labeled gastric cancer cell lines (GLM-4, NCI-N87, and TMK-1) to express CD137. After incubation for 18 h, ADCC following the addition of rhCD137L was measured to assess the initiation of apoptosis. The incubation time was determined according to the time required to observe CD137 expression, with CD137 not expressed on resting NK cells, but reaching a peak at between 18 h and 24 h after incubation. As expected, trastuzumab alone increased NK cell cytotoxicity against GLM-4 and NCI-N87 cells. Furthermore, the combination with rhCD137L administration induced significantly higher ADCC activity than trastuzumab alone (Fig 2A and 2B). However, there was little cytotoxicity observed against TMK-1 cells in this condition with or without trastuzumab and rhCD137L (Fig 2C). These results indicated that rhCD137-mediated antitumor activity was dependent upon the HER2 expression levels in the tumor cells.

### rhCD137L enhances cytokine secretion of activated NK cells

**Fig 3:**
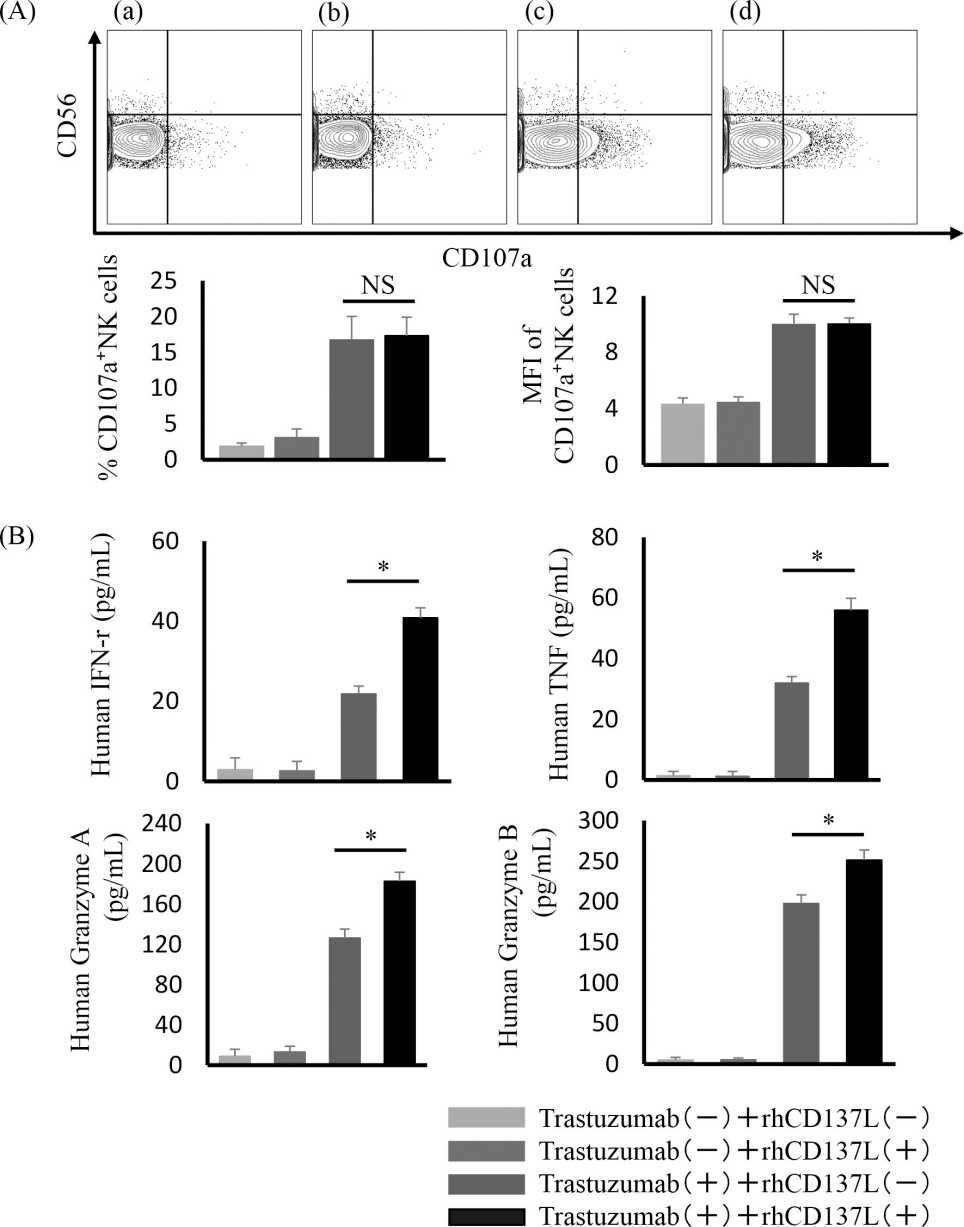
Increased cytokine secretion of trastuzumab-treated NK cells by rhCD137L administration. To investigate NK cell degranulation and cytokine secretion, NK cells were incubated with GLM-4 in the following four conditions: medium alone, trastuzumab (10 ng/mL), rhCD137L (250 ng/mL), or trastuzumab (10 ng/mL) with rhCD137L (250 ng/mL). (A) A representative flow cytometric plot of CD56 and CD107a double staining. Percentage and MFI of CD107a-expressing NK cells from five healthy individuals [*p* = not significant (NS)]. (B) Cytokine secretion (human IFN-γ, TNF, granzyme A, or granzyme B) determined by the cytometric bead array (*p < 0.005). Data are shown as the mean ± SEM.

Another function of activated NK cells was investigated next by measuring CD107a mobilization and secretion of cytokines by incubation with GLM-4 cells. Purified NK cells from five healthy human patients were incubated with GLM-4 in the following four conditions: medium alone, trastuzumab, rhCD137L, or trastuzumab with rhCD137L. Although trastuzumab increased NK cell degranulation compared with medium or rhCD137L alone, CD107a mobilization (Fig 3A) was not observed with the combination of trastuzumab and rhCD137L. Furthermore, another typical activated marker was not upregulated in trastuzumab-treated NK cells treated with rhCD137L compared with trastuzumab alone (S2 Fig). However, rhCD137L significantly increased trastuzumab-induced cytokine secretion (human IFN-γ, TNF, granzyme A, or granzyme B) (Fig 3B).

### rhCD137L increases cetuximab-mediated NK cell cytotoxicity

**Fig 4:**
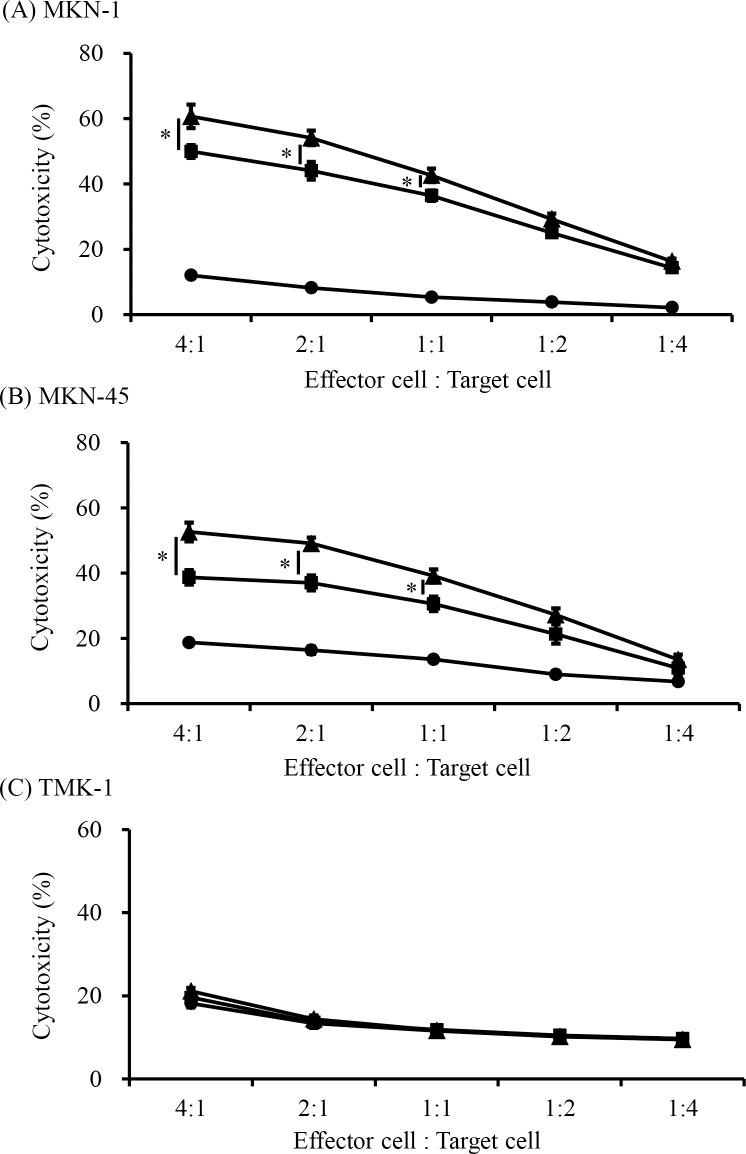
Enhancement of cetuximab-mediated NK cell cytotoxicity by rhCD137L administration. Purified NK cells were analyzed for cytotoxicity with cetuximab (10 ng/mL) plus rhCD137L (250 ng/mL) in a chromium-release assay, as described previously. NK cells were incubated with gastric cell lines expressing high levels of EGFR [(A) MKN-1 and (B) MKN-45] or low levels of EGFR [(C) TMK-1] under the same conditions. Lysis percentages of the target cells cultured with medium alone (circles), cetuximab (squares), or cetuximab and rhCD137L (triangles) according to chromium release at varying effector (NK cells)/target-cell ratios are shown. (A, B, *p < 0.01; C, *p* = not significant). Data are shown as the mean ± SEM.

We hypothesized that enhanced ADCC activity following rhCD137L administration might also be mediated by other tumor-targeting mAbs. Cetuximab is a recombinant human-mouse chimeric mAb against EGFR and exhibits antitumor activity against various cancers [21]. Therefore, we investigated whether cetuximab plus CD137 stimulation could enhance cetuximab-mediated NK cell cytotoxicity and degranulation in gastric cancer cell lines. Similar to results observed involving trastuzumab treatment, cetuximab-coated EGFR-expressing cells induced significant upregulation of CD137 expression and presentation on NK cells. This CD137 induction was dependent upon EGFR expression levels on gastric cancer cells (Spearman correlation test; *p* < 0.001) (S3 Fig), with NK cells promoting cetuximab-mediated cytotoxicity in the EGFR-expressing gastric cancer cell lines MKN-1 and MKN-45. Furthermore, the combination of cetuximab and rhCD137L enhanced ADCC activity to a degree greater than that induced by cetuximab alone (Fig 4A and 4B). However, this efficacy was not observed against gastric cancer cells expressing low levels of EGFR (TMK-1) (Fig 4C). NK cell degranulation was also investigated with MKN-1 cells. Similar to trastuzumab treatment, CD107a mobilization was not observed (Panel A in S4 Fig). Nonetheless, rhCD137L significantly increased cytokine secretion of cetuximab-treated NK cells (Panel B in S4 Fig).

### Trastuzumab plus pertuzumab induces upregulated CD137 expression on human NK cells, and rhCD137L increases dual anti-HER2 mAb-mediated NK cell cytotoxicity

**Fig 5:**
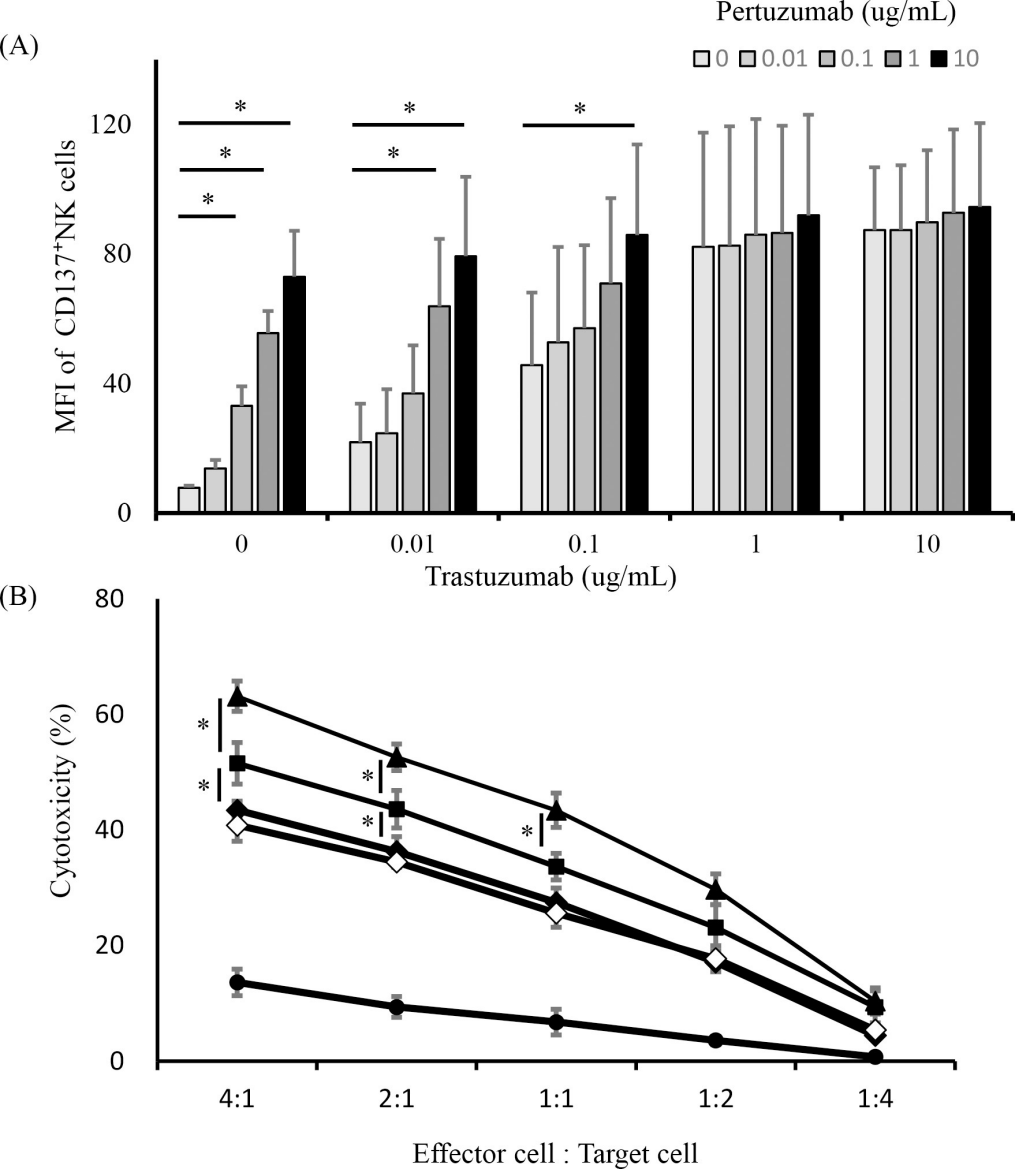
Upregulated CD137 expression and cytotoxicity of NK cells by treatment with a combination of trastuzumab and pertuzumab. (A) To evaluate upregulated CD137 expression in NK cells by treatment with the combination of dual anti-HER2 mAbs that bind different domains, purified NK cells were incubated with a HER2-expressing gastric cancer cell line (GLM-4) in medium containing various trastuzumab and pertuzumab concentrations (0–10 μg/mL) for 24 h. The MFI of CD137-expressing NK cells from three healthy individuals was analyzed by flow cytometry (*p < 0.05). Data are shown as the mean ± SEM. (B) Purified NK cells were analyzed for cytotoxicity in a chromium-release assay. Chromium-labeled GLM-4 cells and NK cells cultured with medium alone (circles), pertuzumab (white diamonds), trastuzumab (black diamonds), pertuzumab and trastuzumab (squares), or dual anti-HER2 mAbs and rhCD137L (triangles) are shown. (*p < 0.01). Data are shown as the mean ± SEM.

Pertuzumab is also a recombinant humanized immunoglobulin mAb that targets a HER2 dimerization domain different from that targeted by trastuzumab. Treatment with a combination of trastuzumab and pertuzumab shows antitumor efficacy against HER2-positive breast cancers via ADCC [22]. Therefore, we hypothesized that the combination of dual anti-HER2 mAbs would activate NK cells more effectively via CD137 stimulation. NK cells from three healthy individuals were cultured with a HER2-overexpressing gastric cancer cell line (GLM4) in the presence of different concentrations of trastuzumab and pertuzumab (0–10 μg/mL) (Fig 5A). We observed that each of the anti-HER2 mAbs increased CD137 expression and presentation on NK cells in a concentration-dependent manner, and that the combination of trastuzumab and pertuzumab additively enhanced CD137 expression, but not synergistically. The function of the activated NK cells was investigated next by cytotoxicity assay in the following five conditions: medium alone, trastuzumab, pertuzumab, trastuzumab and pertuzumab, or trastuzumab and pertuzumab with rhCD137L. The dual anti-HER2 combination enhanced cytotoxicity compared with each mAb alone, and the addition of rhCD137L resulted in further enhancement of ADCC activity (Fig 5B).

### Upregulated CD137 expression on NK cells is stimulated by immobilized IgG subclass

**Fig 6:**
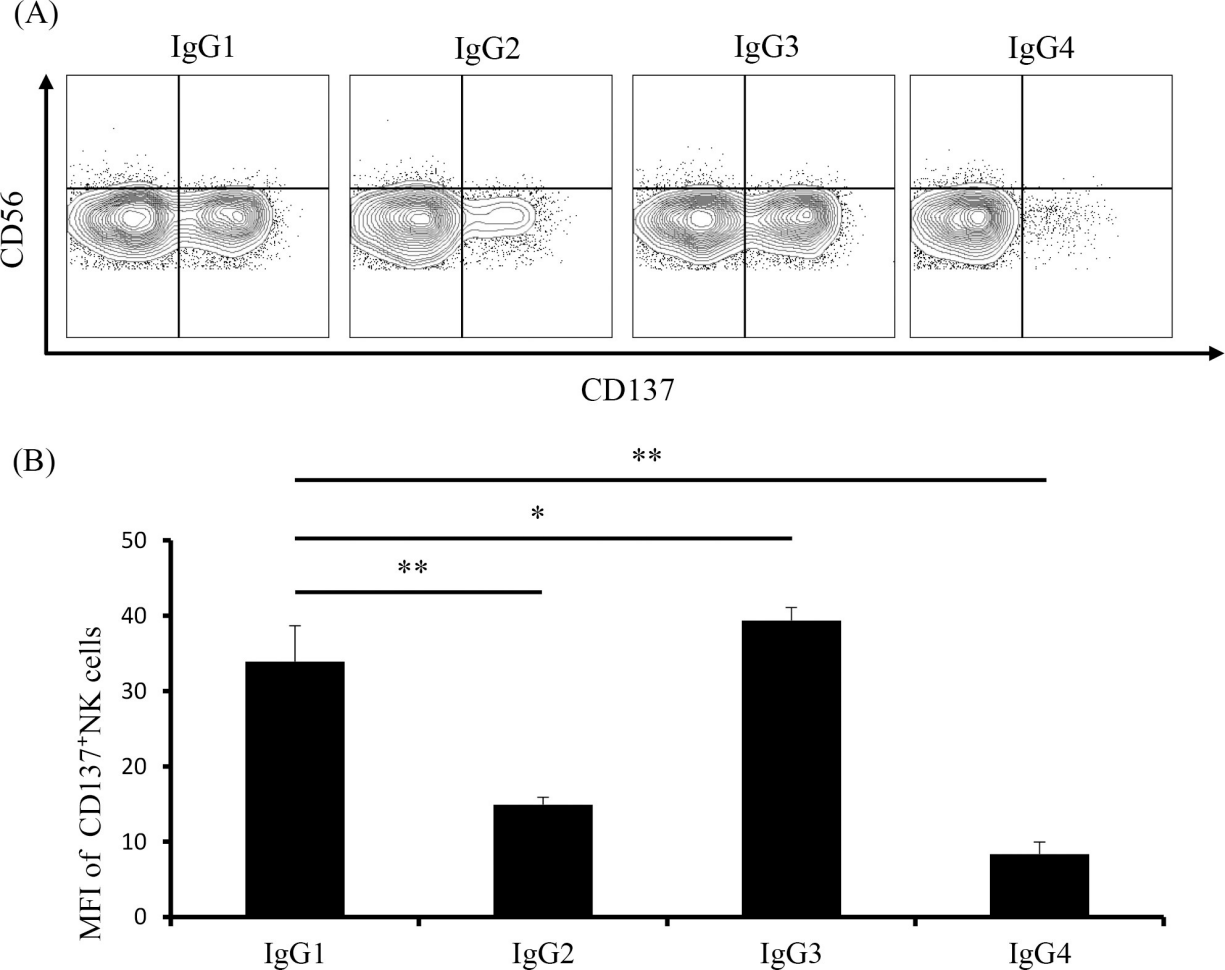
Upregulated CD137 expression induced by incubation with immobilized IgG subclass. Purified NK cells were incubated for 24 h in the presence of immobilized IgG subclass (2 mg/mL), and CD137 expression was measured by flow cytometry. (A) CD137 expression on NK cells from a representative healthy individual after a 24-h culture. Percentages of CD137-expressing NK cells per quadrant are indicated. (B) MFI of CD137-expressing NK cells from five healthy individuals. **p < 0.001; *p = 0.016. Data are shown as the mean ± SEM.

Upregulated CD137 expression occurs following mAb binding to NK cell FcγR [23]. However, it remains unclear whether Fc-FcγR affinity to these mAbs affects CD137 expression in NK cells. We investigated changes in CD137 expression initiated by human IgG subclass, which exhibits different Fc-binding activity. To eliminate the influence of co-stimulatory receptor effects on tumor cells, NK cells were incubated in the presence of immobilized or soluble mAbs in the absence of tumor cells. CD137 expression in NK cells was elevated by immobilized IgG1 mAbs, but not at all by soluble IgG1 mAbs (Panel A in S5 Fig). Subsequent culture of NK cells with various concentrations of immobilized IgG1 to select the most suitable conditions showed that CD137 expression was enhanced in an immobilized-mAb-concentration-dependent manner, reaching a plateau at 4 mg/mL (Panel B in S5 Fig). Based on these results, we investigated CD137 expression in NK cells stimulated by immobilized IgG subclass at 2 mg/mL. Our results showed that IgG1 and IgG3 enhanced CD137 expression in NK cells, with IgG3 resulting in the most significant upregulation, whereas immobilized IgG2 and IgG4 resulted in minimal changes in expression (Fig 6A and 6B).

### CD137 expression in human NK cells derived from patients harboring different single-nucleotide polymorphisms (SNPs)

**Fig 7:**
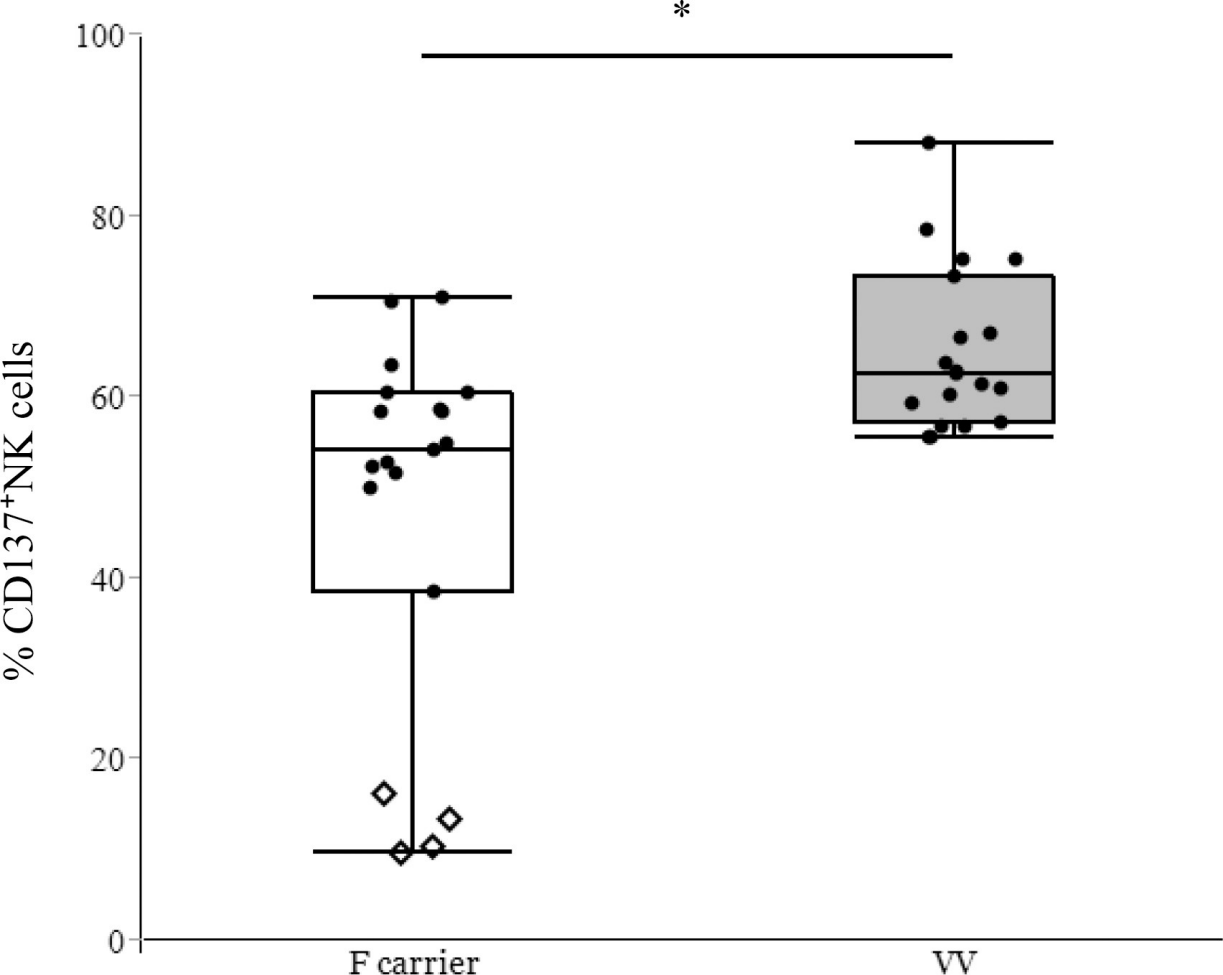
The influence of FcγRIIIa polymorphisms on trastuzumab-induced CD137 expression. Percentage of CD137-expressing NK cells from 38 healthy individuals after a 24-h incubation with trastuzumab-coated gastric cancer cells (GLM-4) exhibiting FcγRIIIa 158 polymorphism. CD137 presentation on NK cells was compared between V/V (*n* = 19) and F carriers (*n* = 19). F carriers were also divided into two groups: VF (*n* = 15, circles) and FF (*n* = 4, diamonds). *p = 0.014. Data are shown as the mean ± SEM.

We then investigated the influence of FcγRIIIA polymorphisms on CD137 expression in NK cells. IgG1 and IgG3 bind more tightly to FcγRIIIA-158 V/V as compared with 158 F/F, thereby increasing effector-cell activity in individuals with FcγRIIIA-158 V/V [24]. Therefore, we examined the relationship between trastuzumab-induced CD137 expression and NK-cell SNPs (Fig 7). A greater increase in CD137 expression was observed among individuals harboring the high-affinity alleles FcγRIIIA-158 V/V as compared with those harboring the low-affinity 158 F (V/F and F/F) alleles. Specifically, F/F individuals (*n* = 4) showed lower levels of CD137 expression in NK cells relative to those observed with other alleles.

## Discussion

The prognosis of patients with advanced gastric cancer remains poor due to the lack of effective chemotherapeutics and tumor-targeted antibodies [9, 25]. Manipulation of co-stimulatory immune molecules represents a new and attractive strategy for improving therapeutic efficacies. Immunomodulatory antibodies, such as CTLA-4-, PD-1-, and PD-L1-specific mAbs, target the patient immune system to overcome immunosuppression induced by tumor cells and generate an antitumor immune response [26]. Clinical data validate mAb-mediated cancer immunotherapy as a valuable therapeutic strategy, and recently, a clinical trial of gastric cancer patients was conducted using an anti-PD-1 mAb, reporting its ability to significantly prolong overall survival of gastric cancer patients and reduce the risk of death by 37% as compared with placebos [27]. In addition to expanding the clinical benefits of this approach, agents designed to target other immunomodulatory pathways are currently under evaluation, including co-stimulatory molecules, such as CD134 and CD137 [28]. In this study, we hypothesized that CD137 stimulation would enhance the antitumor activity of NK cells against gastric cancer cells. To test this hypothesis, we first confirmed that trastuzumab- or cetuximab-coated gastric cancer cells were capable of inducing CD137 expression in NK cells, followed by confirmation that CD137 stimulation using rhCD137L enhanced NK cell cytotoxicity and the release of cytokines toward mAb-coated tumor cells. Moreover, we demonstrated that CD137 expression was dependent upon differences in IgG subclass and FcγRIIIA polymorphisms.

CD137 is an inducible and co-stimulatory molecule expressed on activated CD4 and CD8 T cells. The majority of previous work focused on CD137 stimulation to increase T cell proliferation and survival [29], and preclinical activity subsequently motivated research into anti-CD137 antibody monotherapy [29–31]. However, CD137 expression is also observed on other functionally significant immune cells, including NK cells, monocytes, dendritic cells, and nonhematopoietic cells [32, 33]. Previous studies demonstrated that CD137 stimulation of NK cells enhances mAb-mediated ADCC functions and works in synergy with anti-CD20, anti-HER2, and anti-EGFR mAbs in xenotransplant murine models of lymphoma, breast, colon, and head and neck cancer [16–18].

Here, we demonstrated that the contact of NK cells with trastuzumab-bound, HER2-expressing gastric cancer cells resulted in significant CD137 expression that directly correlated with the levels of HER2 expression on tumor cells. Our findings supported previous results using immobilized IgG1 or rituximab-coated lymphoma cells to induce CD137 expression [16, 23]. Moreover, after upregulation of CD137 expression in NK cells, we showed that stimulation of this target with rhCD137L enhanced NK cell activity, as measured by cytotoxicity and release of cytokines, toward trastuzumab-bound tumor cells. Although the study entailing trastuzumab treatment for gastric cancer showed a survival benefit with trastuzumab plus chemotherapy in HER2-positive gastric cancer [9], further synergistic effects could be expected by the combined use of CD137 stimulation using molecules such as rhCD137L. These phenomena were also observed in cetuximab experiments. EGFR protein expression and overexpression are reported in 43% and 11% of gastric cancer patients, respectively [34], and EGFR overexpression is a significant predictor of poor survival in gastric cancer patients; however, a randomized phase III trial using cetuximab failed to show a significant improvement in patient survival [35]. Among various mechanisms of resistance to anti-EGFR agents, the frequency of RAS mutations, which serve as the major predictive biomarker of anti-EGFR treatments in colorectal cancer, was found to be low in gastric cancer patients [36]. The search for alternative resistance mechanisms to anti-EGFR agents is currently underway, although these mechanisms do not confer resistance to ADCC. Our results showed that enhancing ADCC along with CD137 stimulation might offer an opportunity to overcome tumor resistance to cetuximab treatment.

We also demonstrated that combination treatment of HER2-expressing gastric cancer cells with trastuzumab and pertuzumab additively enhanced CD137 expression in NK cells, followed by confirmation that CD137 stimulation using rhCD137L enhanced NK cell cytotoxicity toward mAb-coated tumor cells. These combination treatments improved progression-free and overall survival in patients with HER2-positive metastatic breast cancer [22]. Furthermore, combination therapy showed enhanced antitumor activity in preclinical studies of a HER2-positive human gastric cancer xenograft model [37] and is being tested for patients with HER2-positive gastric cancer as part of an international phase III trial [38]. Because trastuzumab and pertuzumab differ in the HER2-binding sites, it was considered that the total number of Fc-binding sites increased significantly, and CD137 expression in NK cells would be enhanced. Other dual anti-HER2 combinations (trastuzumab and lapatinib) have also been found to enhance ADCC in a synergistic fashion [39, 40]. Higher efficacy might be obtained by combining CD137 stimulation with those different targeting agents.

In this study, we demonstrated that IgG1 and IgG3 in the IgG subclass induced more efficient CD137 expression in NK cells. Human IgG is classified into four subclasses: IgG1, IgG2, IgG3, and IgG4. These subclasses differ in their affinity for FcγRIIIA on NK cells, and effector functions, such as ADCC activity, are higher in the presence of IgG1 and IgG3 relative to other classes. Therefore, most therapeutic antibodies that require high levels of effector function and stability *in vivo* were developed in the form of the human IgG1 subclass. Because ADCC activity is exerted by binding to FcγRIIIA, it is possible to enhance this activity by increasing the affinity for FcγRIIIA. Several approaches to enhance this affinity have been reported, including the introduction of amino acid mutations into heavy chain regions [41, 42] or through glycol modifications in Fc-linked oligosaccharides [43]. Our results indicated that the affinity between the Fc portion of IgG and FcγRIIIA located on NK cells was directly associated with CD137 expression, and that these approaches are expected to increase CD137 expression in NK cells.

Previous studies reported that an FcγRIIIa polymorphism at position 158 is associated with the therapeutic efficacy of tumor-targeting mAbs [44–46], and we previously reported that this polymorphism was found in patients predisposed to infectious complications following liver transplantation [20]. In this study, CD137 expression in NK cells was also affected by this polymorphism, and high affinity for FcγRIIIa-158 (V/V) induced greater CD137 expression as compared with that observed in other genotypes. Our results associated with gastric cancer cell lines support previous results related to breast cancer and colorectal cancer [17, 18]. FcγRIIIa polymorphisms depend upon the stage and type of disease or the race of the patients, with East Asian subjects exhibiting a relatively higher frequency of the FcγRIIIa-158 (V/V) genotype than Caucasians [47, 48]. The distribution of FcγRIIIa polymorphisms in gastric cancer patients is unclear, although this disease is more common in East Asian populations. Therefore, changes in CD137 expression due to differences in FcγRIIIa polymorphisms might be more useful as treatment targets and biomarkers in gastric cancer. Currently, a clinical trial is underway to identify differences in FcγRIIIa polymorphisms and CD137 expression in NK cells as predictors for personalized cancer therapy [49], which would also be considered useful information for gastric cancer treatment.

We acknowledge several limitations in this study. First, we used rhCD137L for stimulating activation of NK cells instead of agonist CD137-targeting antibodies. Agonist CD137 mAbs are the most straightforward and extensively studied modality for activating CD137; however, agonistic mAbs can be associated with severe toxicity, such as hepatitis and inflammatory cytokine production, arising from non-specific and systemic activation of lymphocytes. Previous studies demonstrated that a soluble CD137L, a chimeric form created by fusing the extracellular functional domain of CD137L to a modified form of core streptavidin, exhibited improved efficacy and safety as compared with an agonist antibody [50]. These natural co-stimulatory ligands might represent compelling alternative agonists for CD137-mAb-mediated stimulation for tumor immunotherapy. Second, we focused on ADCC mediated by human NK cells. The effects of CD137 stimulation on non-NK cells will be important for clinical applications. As suggested by previous studies, trastuzumab- and cetuximab-induced cytotoxicity might trigger an adaptive immune response, which is improved by CD137 stimulation of activated T cells, B cells, and dendritic cells [17, 18]. Furthermore, the heterogeneity of gastric cancers complicates the innate immune microenvironment. To reveal the *in vivo* effects of CD137 stimulation on different forms of gastric cancer, our future work will investigate the three-dimensional microenvironment using organoid techniques.

## Conclusions

The results of our study suggested that CD137 stimulation enhanced the anti-gastric-cancer effects of trastuzumab and cetuximab, and that CD137 expression was dependent upon mAb-binding affinity with Fc structures and FcγRIIIA polymorphisms. Our results supported those reported previously and indicated possible applicability to gastric cancer. These results provided insight into additional avenues for exploiting the power of NK cells for gastric cancer immunotherapy.

## Supporting information

S1 Fig CD137L and CD137 receptor expression in gastric cancer cell lines. Gastric cancer cell lines were analyzed for CD137L and CD137 receptor expression by flow cytometry. (A) CD137L expression in gastric cancer cell lines. (B) CD137 receptor expression in gastric cancer cell lines.
(TIF)

S2 Fig Phenotype of trastuzumab- and rhCD137L-treated NK cells. MFI of activated marker-expressing NK cells from three healthy individuals. *p* = not significant (NS). Data are shown as the mean ± SEM.
(TIF)

S3 Fig Upregulated CD137 expression in NK cells following incubation with gastric cancer cells and cetuximab. NK cells from healthy individuals were analyzed for CD137 expression after a 24-h culture with gastric cancer cell lines and cetuximab. (A) EGFR expression in gastric cancer cell lines (MKN-1, MKN-45, and TMK-1). (B) CD137 expression in NK cells derived from a representative healthy individual after a 24-h culture with the respective gastric cancer cell lines in the presence of cetuximab.
(TIF)

S4 Fig Increased cytokine secretion of cetuximab-treated NK cells by rhCD137L administration. (A) A representative flow cytometric plot of CD56 and CD107a double staining. Percentage and MFI of CD107a-expressing NK cells from five healthy individuals [*p* = not significant (NS)]. (B) Cytokine secretion (human IFN-γ, TNF, granzyme A, or granzyme B) as determined by cytometric bead array (*p < 0.005). Data are shown as the mean ± SEM.
(TIF)

S5 Fig Upregulated CD137 expression in NK cells incubated with immobilized mAbs. NK cells were cultured in the presence of either immobilized or soluble IgG1 mAbs at various concentrations. Control wells (immobilized IgG1 mAb: 0 μg/mL) were pre-coated overnight with RPMI supplemented with 10% FBS. (A) CD137 expression in NK cells obtained from a representative healthy individual after a 24-h culture. (B) Percentage of CD137-expressing NK cells derived from five healthy individuals and incubated with various concentrations of immobilized IgG1.
(TIF)

## References

[1] Ferlay J, Soerjomataram I, Dikshit R, Eser S, Mathers C, Rebelo M, et al Cancer incidence and mortality worldwide: sources, methods and major patterns in GLOBOCAN 2012. Int J Cancer. 2015;136(5):E359–386. 10.1002/ijc.29210 25220842

[2] Kamangar F, Dores GM, Anderson WF. Patterns of cancer incidence, mortality, and prevalence across five continents: defining priorities to reduce cancer disparities in different geographic regions of the world. J Clin Oncol. 2006;24(14):2137–2150. 10.1200/JCO.2005.05.2308 16682732

[3] Janunger KG, Hafstrom L, Glimelius B. Chemotherapy in gastric cancer: a review and updated meta-analysis. Eur J Surg. 2002;168(11):597–608. 12699095 10.1080/11024150201680005

[4] Van Cutsem E, Moiseyenko VM, Tjulandin S, Majlis A, Constenla M, Boni C, et al Phase III study of docetaxel and cisplatin plus fluorouracil compared with cisplatin and fluorouracil as first-line therapy for advanced gastric cancer: a report of the V325 Study Group. J Clin Oncol. 2006;24(31):4991–4997. 10.1200/JCO.2006.06.8429 17075117

[5] Koizumi W, Narahara H, Hara T, Takagane A, Akiya T, Takagi M, et al S-1 plus cisplatin versus S-1 alone for first-line treatment of advanced gastric cancer (SPIRITS trial): a phase III trial. Lancet Oncol. 2008;9(3):215–221. 10.1016/S1470-2045(08)70035-4 18282805

[6] Varchetta S, Gibelli N, Oliviero B, Nardini E, Gennari R, Gatti G, et al Elements related to heterogeneity of antibody-dependent cell cytotoxicity in patients under trastuzumab therapy for primary operable breast cancer overexpressing Her2. Cancer Res. 2007;67(24):11991–11999. 10.1158/0008-5472.CAN-07-2068 18089830

[7] Gennari R, Menard S, Fagnoni F, Ponchio L, Scelsi M, Tagliabue E, et al Pilot study of the mechanism of action of preoperative trastuzumab in patients with primary operable breast tumors overexpressing HER2. Clin Cancer Res. 2004;10(17):5650–5655. 10.1158/1078-0432.CCR-04-0225 15355889

[8] Clynes RA, Towers TL, Presta LG, Ravetch JV. Inhibitory Fc receptors modulate in vivo cytotoxicity against tumor targets. Nat Med. 2000;6(4):443–446. 10.1038/74704 10742152

[9] Bang YJ, Van Cutsem E, Feyereislova A, Chung HC, Shen L, Sawaki A, et al Trastuzumab in combination with chemotherapy versus chemotherapy alone for treatment of HER2-positive advanced gastric or gastro-oesophageal junction cancer (ToGA): a phase 3, open-label, randomised controlled trial. Lancet. 2010;376(9742):687–697. 10.1016/S0140-6736(10)61121-X 20728210

[10] Matsusaka S, Nashimoto A, Nishikawa K, Miki A, Miwa H, Yamaguchi K, et al Clinicopathological factors associated with HER2 status in gastric cancer: results from a prospective multicenter observational cohort study in a Japanese population (JFMC44-1101). Gastric Cancer. 2016;19(3):839–851. 10.1007/s10120-015-0518-8 26265390 PMC4906061

[11] Adair RA, Roulstone V, Scott KJ, Morgan R, Nuovo GJ, Fuller M, et al Cell carriage, delivery, and selective replication of an oncolytic virus in tumor in patients. Sci Transl Med. 2012;4(138):138ra177. 10.1126/scitranslmed.3003578 PMC3893925 22700953

[12] Ming Lim C, Stephenson R, Salazar AM, Ferris RL. TLR3 agonists improve the immunostimulatory potential of cetuximab against EGFR+ head and neck cancer cells. Oncoimmunology. 2013;2(6):e24677 10.4161/onci.24677 23894722 PMC3716757

[13] Peipp M, Lammerts van Bueren JJ, Schneider-Merck T, Bleeker WW, Dechant M, Beyer T, et al Antibody fucosylation differentially impacts cytotoxicity mediated by NK and PMN effector cells. Blood. 2008;112(6):2390–2399. 10.1182/blood-2008-03-144600 18566325

[14] McDermott DF, Regan MM, Clark JI, Flaherty LE, Weiss GR, Logan TF, et al Randomized phase III trial of high-dose interleukin-2 versus subcutaneous interleukin-2 and interferon in patients with metastatic renal cell carcinoma. J Clin Oncol. 2005;23(1):133–141. 10.1200/JCO.2005.03.206 15625368

[15] Gollob JA, Mier JW, Veenstra K, McDermott DF, Clancy D, Clancy M, et al Phase I trial of twice-weekly intravenous interleukin 12 in patients with metastatic renal cell cancer or malignant melanoma: ability to maintain IFN-gamma induction is associated with clinical response. Clin Cancer Res. 2000;6(5):1678–1692. 10815886

[16] Kohrt HE, Houot R, Goldstein MJ, Weiskopf K, Alizadeh AA, Brody J, et al CD137 stimulation enhances the antilymphoma activity of anti-CD20 antibodies. Blood. 2011;117(8):2423–2432. 10.1182/blood-2010-08-301945 21193697 PMC3062409

[17] Kohrt HE, Houot R, Weiskopf K, Goldstein MJ, Scheeren F, Czerwinski D, et al Stimulation of natural killer cells with a CD137-specific antibody enhances trastuzumab efficacy in xenotransplant models of breast cancer. J Clin Invest. 2012;122(3):1066–1075. 10.1172/JCI61226 22326955 PMC3287235

[18] Kohrt HE, Colevas AD, Houot R, Weiskopf K, Goldstein MJ, Lund P, et al Targeting CD137 enhances the efficacy of cetuximab. J Clin Invest. 2014;124(6):2668–2682. 10.1172/JCI73014 24837434 PMC4089447

[19] Kajitani K, Tanaka Y, Arihiro K, Kataoka T, Ohdan H. Mechanistic analysis of the antitumor efficacy of human natural killer cells against breast cancer cells. Breast Cancer Res Treat. 2012;134(1):139–155. 10.1007/s10549-011-1944-x 22261932

[20] Shimizu S, Tanaka Y, Tazawa H, Verma S, Onoe T, Ishiyama K, et al Fc-Gamma receptor polymorphisms predispose patients to infectious complications after liver transplantation. Am J Transplant. 2016;16(2):625–633. 10.1111/ajt.13492 26517570

[21] Liu L, Cao Y, Tan A, Liao C, Gao F. Cetuximab-based therapy versus non-cetuximab therapy for advanced cancer: a meta-analysis of 17 randomized controlled trials. Cancer Chemother Pharmacol. 2010;65(5):849–861. 10.1007/s00280-009-1090-x 19680654

[22] Swain SM, Kim SB, Cortes J, Ro J, Semiglazov V, Campone M, et al Pertuzumab, trastuzumab, and docetaxel for HER2-positive metastatic breast cancer (CLEOPATRA study): overall survival results from a randomised, double-blind, placebo-controlled, phase 3 study. Lancet Oncol. 2013;14(6):461–471. 10.1016/S1470-2045(13)70130-X 23602601 PMC4076842

[23] Lin W, Voskens CJ, Zhang X, Schindler DG, Wood A, Burch E, et al Fc-dependent expression of CD137 on human NK cells: insights into "agonistic" effects of anti-CD137 monoclonal antibodies. Blood. 2008;112(3):699–707. 10.1182/blood-2007-11-122465 18519814 PMC2481534

[24] Bruhns P. Properties of mouse and human IgG receptors and their contribution to disease models. Blood. 2012;119(24):5640–5649. 10.1182/blood-2012-01-380121 22535666

[25] Fuchs CS, Tomasek J, Yong CJ, Dumitru F, Passalacqua R, Goswami C, et al Ramucirumab monotherapy for previously treated advanced gastric or gastro-oesophageal junction adenocarcinoma (REGARD): an international, randomised, multicentre, placebo-controlled, phase 3 trial. Lancet. 2014;383(9911):31–39. 10.1016/S0140-6736(13)61719-5 24094768

[26] Postow MA, Callahan MK, Wolchok JD. Immune checkpoint blockade in cancer therapy. J Clin Oncol. 2015;33(17):1974–1982. 10.1200/JCO.2014.59.4358 25605845 PMC4980573

[27] Kang YK, Satoh T, Ryu MH, Chao Y, Kato K, Chung HC, et al Nivolumab (ONO4538/BMS936558) as salvage treatment after second or later line chemotherapy for advanced gastric or gastro-esophageal junction cancer (AGC): a double-blinded, randomized, phase III trial. J Clin Oncol. 2017;35(suppl 4S; abstract 2).

[28] Vilgelm AE, Johnson DB, Richmond A. Combinatorial approach to cancer immunotherapy: strength in numbers. J Leukoc Biol. 2016;100(2):275–290. 10.1189/jlb.5RI0116-013RR 27256570 PMC6608090

[29] Melero I, Hervas-Stubbs S, Glennie M, Pardoll DM, Chen L. Immunostimulatory monoclonal antibodies for cancer therapy. Nat Rev Cancer. 2007;7(2):95–106. 10.1038/nrc2051 17251916

[30] Murillo O, Arina A, Hervas-Stubbs S, Gupta A, McCluskey B, Dubrot J, et al Therapeutic antitumor efficacy of anti-CD137 agonistic monoclonal antibody in mouse models of myeloma. Clin Cancer Res. 2008;14(21):6895–6906. 10.1158/1078-0432.CCR-08-0285 18980984 PMC2583963

[31] Takeda K, Kojima Y, Uno T, Hayakawa Y, Teng MW, Yoshizawa H, et al Combination therapy of established tumors by antibodies targeting immune activating and suppressing molecules. J Immunol. 2010;184(10):5493–5501. 10.4049/jimmunol.0903033 20400706

[32] Wilcox RA, Chapoval AI, Gorski KS, Otsuji M, Shin T, Flies DB, et al Cutting edge: expression of functional CD137 receptor by dendritic cells. J Immunol. 2002;168(9):4262–4267. 11970964 10.4049/jimmunol.168.9.4262

[33] Narazaki H, Zhu Y, Luo L, Zhu G, Chen L. CD137 agonist antibody prevents cancer recurrence: contribution of CD137 on both hematopoietic and nonhematopoietic cells. Blood. 2010;115(10):1941–1948. 10.1182/blood-2008-12-192591 20068221 PMC2837330

[34] Atmaca A, Werner D, Pauligk C, Steinmetz K, Wirtz R, Altmannsberger HM, et al The prognostic impact of epidermal growth factor receptor in patients with metastatic gastric cancer. BMC Cancer. 2012;12:524 10.1186/1471-2407-12-524 23153332 PMC3529672

[35] Lordick F, Kang YK, Chung HC, Salman P, Oh SC, Bodoky G, et al Capecitabine and cisplatin with or without cetuximab for patients with previously untreated advanced gastric cancer (EXPAND): a randomised, open-label phase 3 trial. Lancet Oncol. 2013;14(6):490–499. 10.1016/S1470-2045(13)70102-5 23594786

[36] Moehler M, Mueller A, Trarbach T, Lordick F, Seufferlein T, Kubicka S, et al Cetuximab with irinotecan, folinic acid and 5-fluorouracil as first-line treatment in advanced gastroesophageal cancer: a prospective multi-center biomarker-oriented phase II study. Ann Oncol. 2011;22(6):1358–1366. 10.1093/annonc/mdq591 21119032

[37] Yamashita-Kashima Y, Iijima S, Yorozu K, Furugaki K, Kurasawa M, Ohta M, et al Pertuzumab in combination with trastuzumab shows significantly enhanced antitumor activity in HER2-positive human gastric cancer xenograft models. Clin Cancer Res. 2011;17(15):5060–5070. 10.1158/1078-0432.CCR-10-2927 21700765

[38] Hoff P, Tabernero J, Shen L, Ohtsu A, Yu R, Szado T, et al Pertuzumab, trastuzumab and chemotherapy in HER2-positive metastatic gastric or gastro-esophageal junction cancer: an international phase III study (JACOB). Annals of Oncology. 2013;24(suppl 4):iv 67.

[39] Scaltriti M, Verma C, Guzman M, Jimenez J, Parra JL, Pedersen K, et al Lapatinib, a HER2 tyrosine kinase inhibitor, induces stabilization and accumulation of HER2 and potentiates trastuzumab-dependent cell cytotoxicity. Oncogene. 2009;28(6):803–814. 10.1038/onc.2008.432 19060928

[40] Musolino A, Naldi N, Dieci MV, Zanoni D, Rimanti A, Boggiani D, et al Immunoglobulin G fragment C receptor polymorphisms and efficacy of preoperative chemotherapy plus trastuzumab and lapatinib in HER2-positive breast cancer. Pharmacogenomics J. 2016;16(5):472–477. 10.1038/tpj.2016.51 27378608

[41] Stavenhagen JB, Gorlatov S, Tuaillon N, Rankin CT, Li H, Burke S, et al Fc optimization of therapeutic antibodies enhances their ability to kill tumor cells in vitro and controls tumor expansion in vivo via low-affinity activating Fcgamma receptors. Cancer Res. 2007;67(18):8882–8890. 10.1158/0008-5472.CAN-07-0696 17875730

[42] Zalevsky J, Leung IW, Karki S, Chu SY, Zhukovsky EA, Desjarlais JR, et al The impact of Fc engineering on an anti-CD19 antibody: increased Fcgamma receptor affinity enhances B-cell clearing in nonhuman primates. Blood. 2009;113(16):3735–3743. 10.1182/blood-2008-10-182048 19109559

[43] Yamane-Ohnuki N, Kinoshita S, Inoue-Urakubo M, Kusunoki M, Iida S, Nakano R, et al Establishment of FUT8 knockout Chinese hamster ovary cells: an ideal host cell line for producing completely defucosylated antibodies with enhanced antibody-dependent cellular cytotoxicity. Biotechnol Bioeng. 2004;87(5):614–622. 10.1002/bit.20151 15352059

[44] Cartron G, Dacheux L, Salles G, Solal-Celigny P, Bardos P, Colombat P, et al Therapeutic activity of humanized anti-CD20 monoclonal antibody and polymorphism in IgG Fc receptor FcgammaRIIIa gene. Blood. 2002;99(3):754–758. 11806974 10.1182/blood.v99.3.754

[45] Tamura K, Shimizu C, Hojo T, Akashi-Tanaka S, Kinoshita T, Yonemori K, et al FcgammaR2A and 3A polymorphisms predict clinical outcome of trastuzumab in both neoadjuvant and metastatic settings in patients with HER2-positive breast cancer. Ann Oncol. 2011;22(6):1302–1307. 10.1093/annonc/mdq585 21109570

[46] Bibeau F, Lopez-Crapez E, Di Fiore F, Thezenas S, Ychou M, Blanchard F, et al Impact of Fc{gamma}RIIa-Fc{gamma}RIIIa polymorphisms and KRAS mutations on the clinical outcome of patients with metastatic colorectal cancer treated with cetuximab plus irinotecan. J Clin Oncol. 2009;27(7):1122–1129. 10.1200/JCO.2008.18.0463 19164213

[47] Chong KT, Ho WF, Koo SH, Thompson P, Lee EJ. Distribution of the FcgammaRIIIa 176 F/V polymorphism amongst healthy Chinese, Malays and Asian Indians in Singapore. Br J Clin Pharmacol. 2007;63(3):328–332. 10.1111/j.1365-2125.2006.02771.x 16981896 PMC2000731

[48] Oboshi W, Watanabe T, Matsuyama Y, Kobara A, Yukimasa N, Ueno I, et al The influence of NK cell-mediated ADCC: structure and expression of the CD16 molecule differ among FcgammaRIIIa-V158F genotypes in healthy Japanese subjects. Hum Immunol. 2016;77(2):165–171. 10.1016/j.humimm.2015.11.001 26582002

[49] Makkouk A, Sundaram V, Chester C, Chang S, Colevas AD, Sunwoo JB, et al Characterizing CD137 upregulation on NK cells in patients receiving monoclonal antibody therapy. Ann Oncol. 2017;28(2):415–420. 10.1093/annonc/mdw570 27831501 PMC6246233

[50] Schabowsky RH, Elpek KG, Madireddi S, Sharma RK, Yolcu ES, Bandura-Morgan L, et al A novel form of 4-1BBL has better immunomodulatory activity than an agonistic anti-4-1BB Ab without Ab-associated severe toxicity. Vaccine. 2009;28(2):512–522. 10.1016/j.vaccine.2009.09.127 19836479 PMC2805442

