# Supplementary figures and images for: Stimulation of natural killer cells with rhCD137 ligand enhances tumor-targeting antibody efficacy in gastric cancer
Source: PLoS One. 2018 Oct 15;13(10):e0204880. doi: 10.1371/journal.pone.0204880 (PMC6188629; doi:10.1371/journal.pone.0204880)

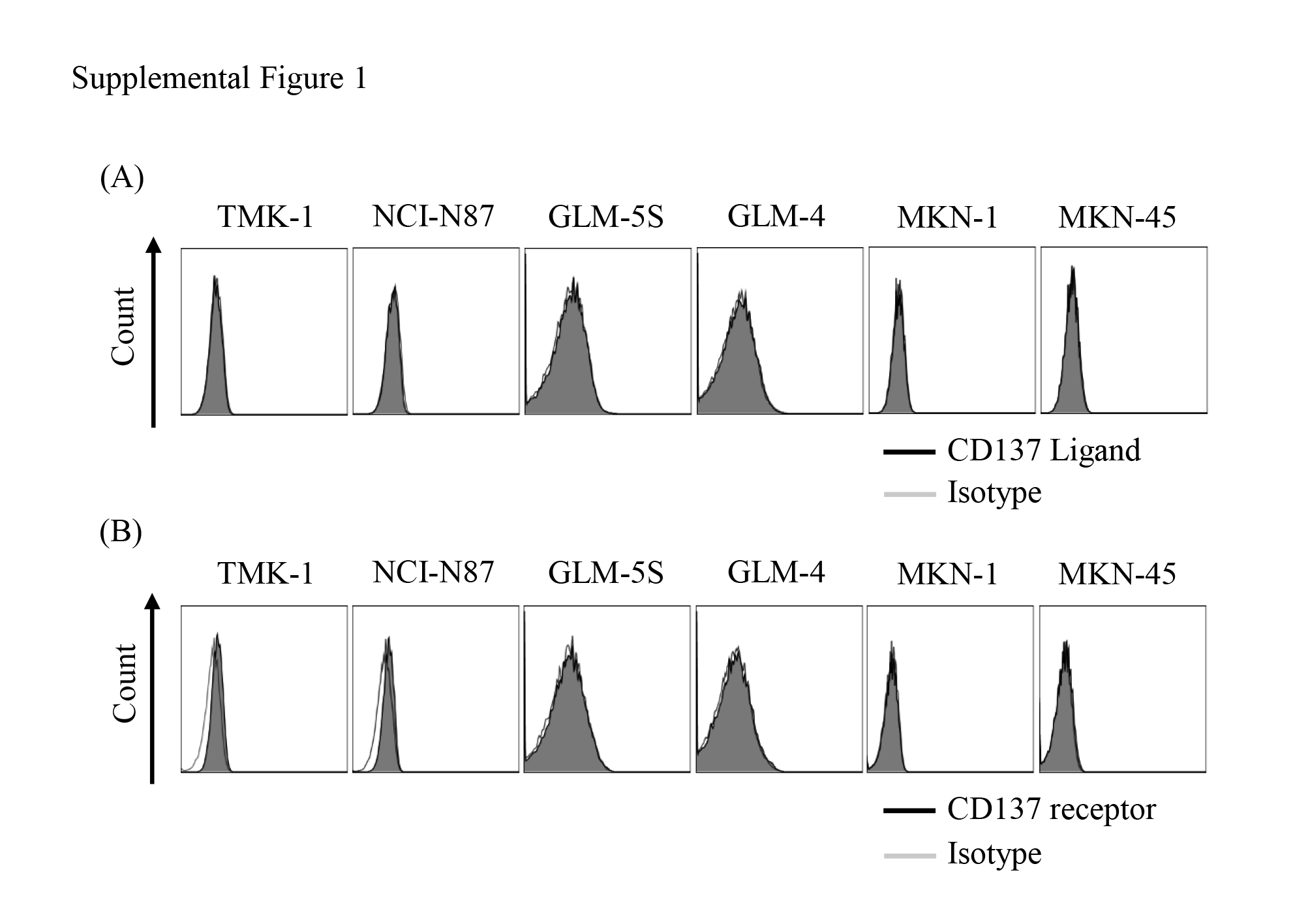

Supplement: S1 Fig — Gastric cancer cell lines were analyzed for CD137L and CD137 receptor expression by flow cytometry. (A) CD137L expression in gastric cancer cell lines. (B) CD137 receptor expression in gastric cancer cell lines. (TIF) [file pone.0204880.s001.tif]

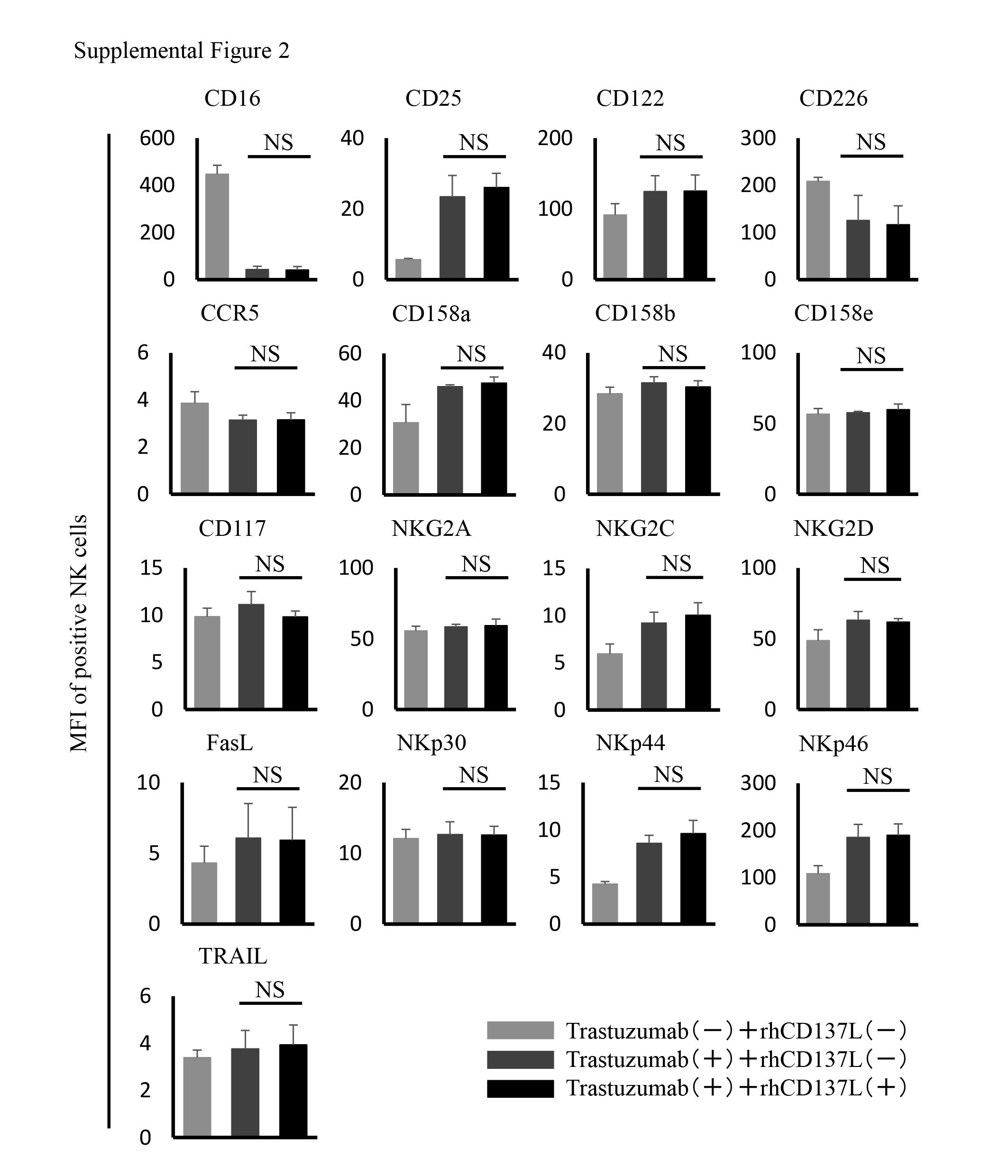

Supplement: S2 Fig — MFI of activated marker-expressing NK cells from three healthy individuals. p = not significant (NS). Data are shown as the mean ± SEM. (TIF) [file pone.0204880.s002.tif]

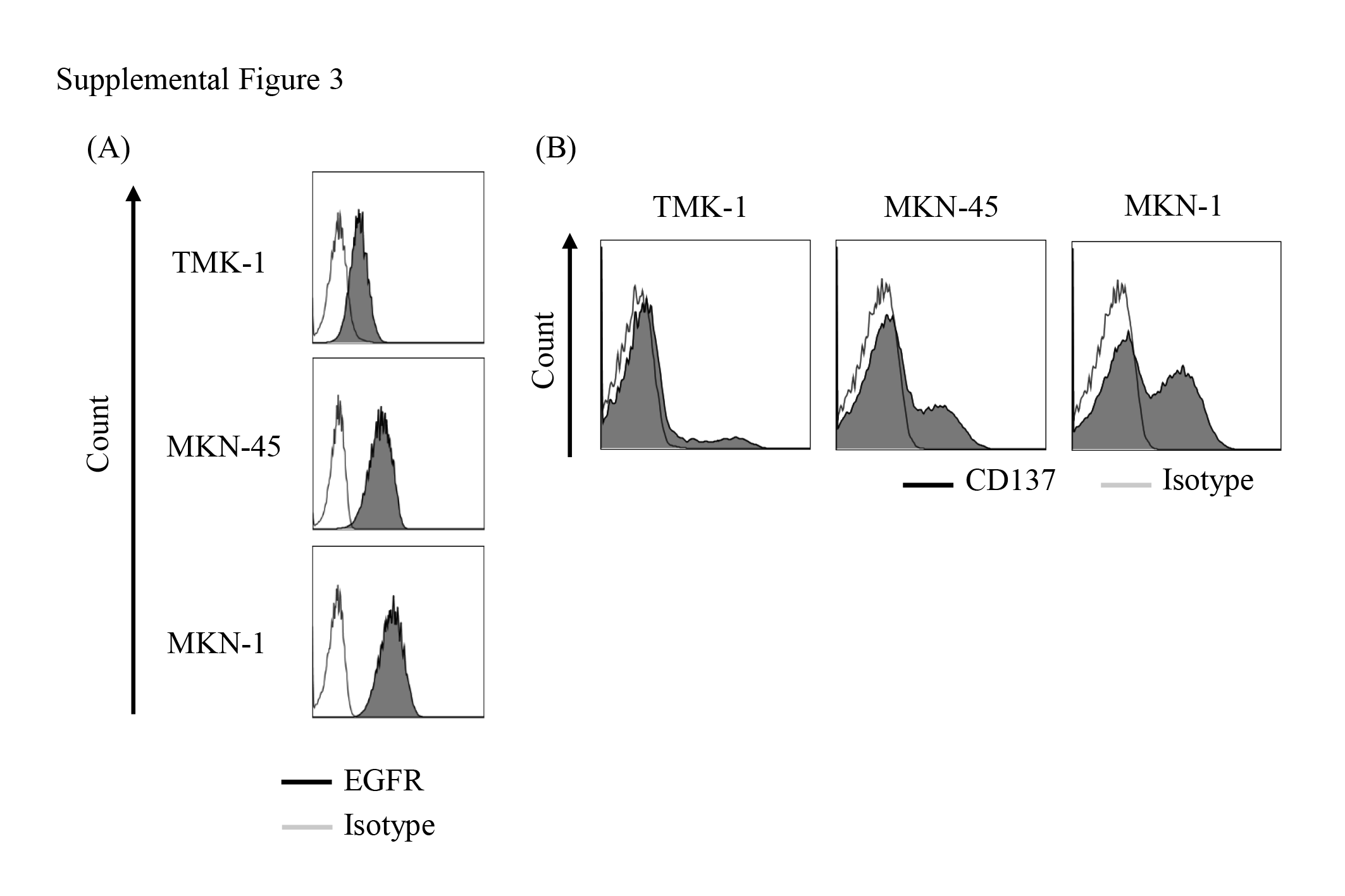

Supplement: S3 Fig — NK cells from healthy individuals were analyzed for CD137 expression after a 24-h culture with gastric cancer cell lines and cetuximab. (A) EGFR expression in gastric cancer cell lines (MKN-1, MKN-45, and TMK-1). (B) CD137 expression in NK cells derived from a representative healthy individual after a 24-h culture with the respective gastric cancer cell lines in the presence of cetuximab. (TIF) [file pone.0204880.s003.tif]

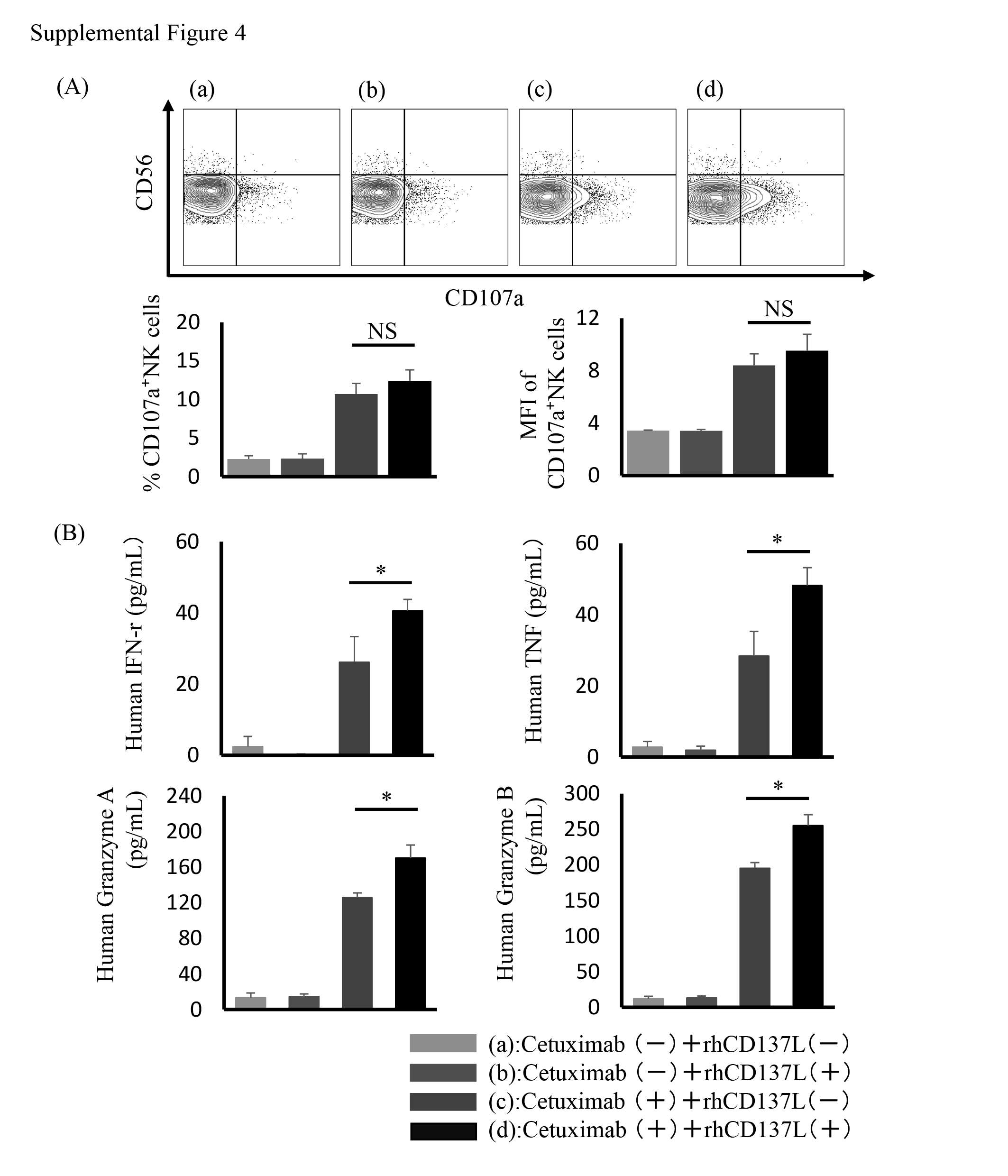

Supplement: S4 Fig — (A) A representative flow cytometric plot of CD56 and CD107a double staining. Percentage and MFI of CD107a-expressing NK cells from five healthy individuals [p = not significant (NS)]. (B) Cytokine secretion (human IFN-γ, TNF, granzyme A, or granzyme B) as determined by cytometric bead array (*p < 0.005). Data are shown as the mean ± SEM. (TIF) [file pone.0204880.s004.tif]

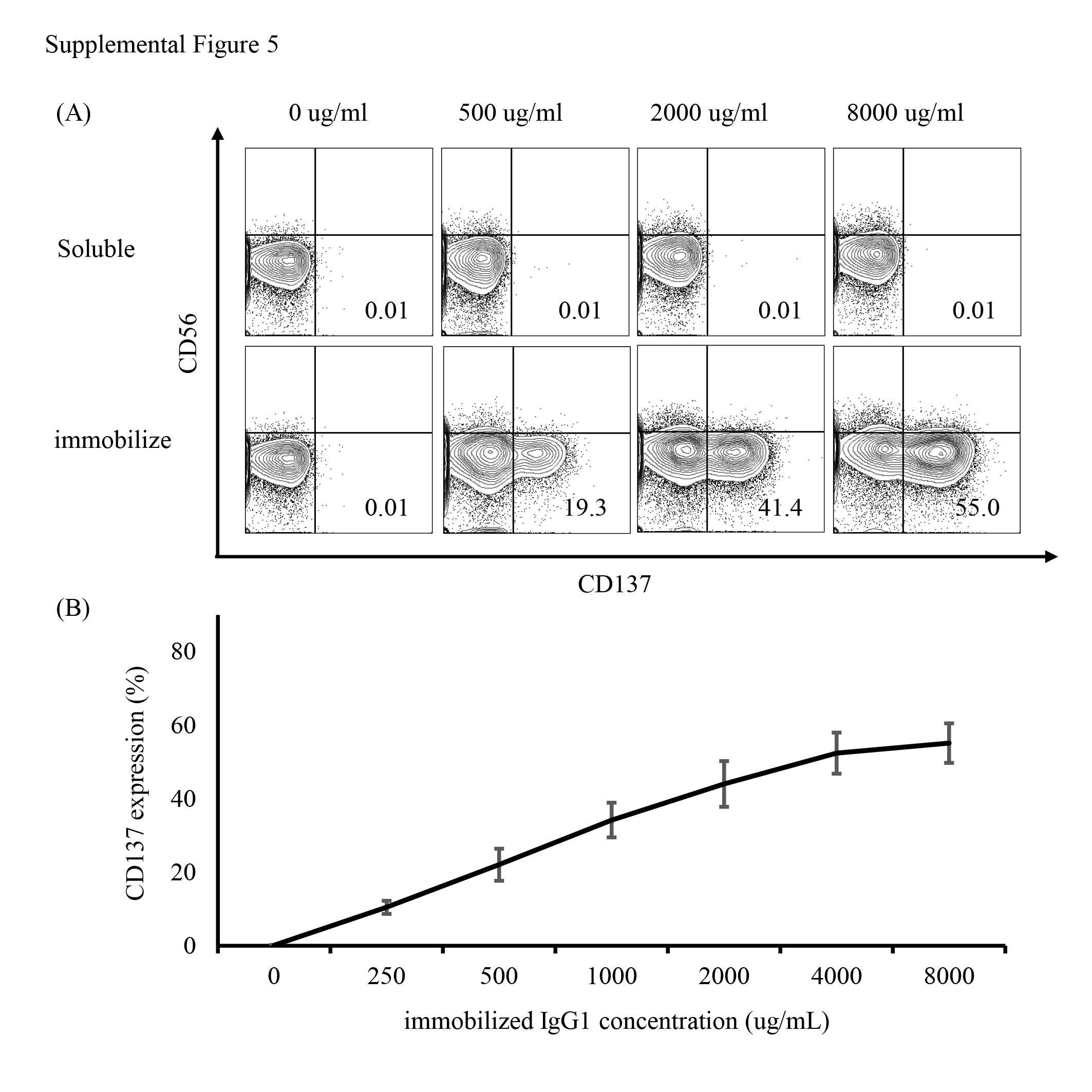

Supplement: S5 Fig — NK cells were cultured in the presence of either immobilized or soluble IgG1 mAbs at various concentrations. Control wells (immobilized IgG1 mAb: 0 μg/mL) were pre-coated overnight with RPMI supplemented with 10% FBS. (A) CD137 expression in NK cells obtained from a representative healthy individual after a 24-h culture. (B) Percentage of CD137-expressing NK cells derived from five healthy individuals and incubated with various concentrations of immobilized IgG1. (TIF) [file pone.0204880.s005.tif]
